# The Metropolitan Water Board and the London Hospitals

**Published:** 1907-06-22

**Authors:** 


### THE METROPOLITAN WATER BOARD AND THE LONDON HOSPITALS.

### A SUBSTANTIAL REDUCTION IN THE COST OF WATER.

The Central Hospital Council for London has taken active steps with a view to secure a reduction in the rates to hospitals for water which the Metropolitan Water Board are seeking power to obtain through their Charges, etc., Bill now before Parliament. Mr. Charles Burt, the late chairman of the Royal Free Hospital, where he rendered most excellent service, has done yeoman service in fighting the battle of the hospitals. Mr. Holland and Mr. Wainwright also gave evidence in support of free water for the London hospitals. An endeavour has been made to secure that all the hospitals, so long as they continue to be occupied or used for the purpose, shall be entitled to a sufficient supply of water free and exempt from the rates and charges authorised under the Water Act. The supply of water to the hospitals, except as to payment, is to be in all respects treated as a supply for domestic purposes under the Act, providing that all expenses incurred in conveying the supply of water from the pipes of the Board to each hospital shall be borne and paid by such hospital.

At the present time the prices for water paid by the hospitals vary from 6d. to 7½d. per 1,000 gallons. These charges represent a cost in the hospitals of London of 1s. 6d. per patient per annum, so that the actual cost to the ratepayers of the gift of free water to the hospitals would represent about £9,000 a year towards the maintenance of the ninety-four hospitals affected. Parliament has already sanctioned the gift of free water to voluntary hospitals in Belfast, in Edinburgh, in Glasgow, and in Leith. Only a nominal charge is made for water at Cork—*i.e.*, 1d. per 1,000 gallons; in Exeter there is a fixed charge of £50 a year; in Liverpool the charge is one guinea per annum for ordinary purposes, with 6d. per 1,000 gallons for water used for lifts and laundry; in Plymouth there is a nominal charge of 1s. per annum. In New York the hospitals are supplied with free water. In Paris and most Continental cities the whole cost of the water for the hospitals is borne by the municipality or the Government. Mr. Burt declares that London treats its hospitals worse than any other city in the world, and the ratepayers make them pay public rates as well as water rates, despite the fact that the Select Committee of the House of Commons in 1900 reported in favour of the hospitals being exempt. Seeing that the ratepayers of London practically pay for the water supplied for the extinction of fires, Mr. Burt holds that the supply of water to hospitals may fairly be treated on the same lines. To do so would mean that the ratepayers would pay for the extinction of disease, which is more important than fire. If the community pay for the latter they may fairly be required to pay for the former. After hearing counsel, the Select Committee of the Houses of Lords and Commons rejected the free-water clause for London hospitals submitted by the Central Hospital Council for London.

Subsequently, the Committee inserted amendments in the Metropolitan Water Board Bill, by which hospitals and sanatoria are brought under the domestic supply rate, namely 5 per cent. on the rateable value of the premises, instead of the compulsory water supply. The effect of this change will be that the cost of water to the hospitals will be in nearly all cases reduced, and will be the means of saving some £5,000 a year in the working expenses of ninety-four hospitals of London. The Bill is to come into operation on April 1, 1908, but we hope when it leaves the Committee and is submitted to the Houses, that in each House some member will move the insertion of the free-water clause for hospitals and divide upon it. It is perfectly clear, seeing that Parliament has already given statutory power to supply free water to the hospitals of Belfast, Edinburgh, and Glasgow, that there is no sufficient reason why a similar power should not be conferred upon the Metropolitan Water Board in the case of the London hospitals.

The following table gives the names of the twenty-one hospitals which are represented on the Central Hospital Council for London. The present charge for water and the new charge under the Bill are shown. This table is very instructive reading for the hospital economist. It well illustrates where great intelligence has been brought to bear on the administration and expenditure of particular hospitals. We are firm believers in the system whereby an economy committee is appointed at each hospital to question, examine, and test every item of expenditure, so as to secure that no money shall be spent, except upon evidence that in every case the best possible article will be supplied at the lowest possible cost.

PRESENT AND PROPOSED NEW CHARGE FOR WATER AT EACH OF THE TWENTY-ONE METROPOLITAN HOSPITALS REPRESENTED ON THE CENTRAL HOSPITAL COUNCIL.

| Hospital | District | Meter Revenue, 1906 | | 5 per cent. on Rateable Value | Rateable Value |
|---|---|---|---|---|---|
| | | Price | Am't | | |
| | | | £ | £ | £ |
| 1. Charing Cross ... ... ... | New River | Scale | 254 | 122 | 2,432 |
| 2. Great Northern ... ... | ,, | ,, | 197 | 44 | 875 |
| 3. Paralysed and Epileptic ... | ,, | ,, | 215 | 101 | 2,026 |
| 4. Royal Free ... ... ... | ,, | ,, | 187 | 73 | 1,457 |
| 5. Royal London Ophthalmic... | ,, | ,, | 70 | 107 | 2,131 |
| 6. Sick Children ... ... ... | ,, | ,, | 150 | 99 | 1,975 |
| 7. West London ... ... ... | West Mid. | ,, | 108 | 35 | 690 |
| 8. Westminster ... ... ... | Chelsea | ,, | 148 | 56 | 1,111 |
| 9. St. George's ... ... ... | ,, | ,, | 352 | 118 | 2,361 |
| 10. Middlesex ... ... ... | West Mid. | Scale | 113* | 86 | 1,724 |
| 11. Guy's ... ... ... ... | Southwark | fixed Scale & 7½d. | 63 | 277 | 5,530 |
| 12. King's ... ... ... ... | New River | 7½d. | 216 | 69 | 1,389 |
| 13. University ... ... ... | ,, | 7½d. | 506 | 75 | 1,500 |
| 14. Seamen's Hospital ... ... | E. London | 7½d. | 138 | 30 | 609 |
| 15. Consumption and Chest ... | Chelsea | 6d. | 334 | 125 | 2,500 |
| 16. St. Bartholomew's ... ... | New River | 6d. | 423 | 59 | 1,184 |
| 17. St. Mary's ... ... ... | Grand Jun. | 6d. | 303 | 75 | 1,500 |
| 18. St. Thomas's ... ... ... | Southwark | 6d.† | 1,034 | 415 | 8,300 |
| 19. East London Children's ... | E. London | 3d. | 45 | 20 | 409 |
| 20. Poplar ... ... ... ... | ,, | 3d. | — | — | — |
| 21. London Hospital proper | ,, | 3d. | 566 | 108 | 2,163 |
| Nurses' Home ... ... | | | | | |
| College and Laundry ... | — | 6d. | — | — | — |
| | | | 5,422 | 2,994 | — |

* £38, £75. † Less 20 per cent.

